# Axotomy-induced HIF-serotonin signalling axis promotes axon regeneration in *C. elegans*

**DOI:** 10.1038/ncomms10388

**Published:** 2016-01-21

**Authors:** Tanimul Alam, Hiroki Maruyama, Chun Li, Strahil Iv. Pastuhov, Paola Nix, Michael Bastiani, Naoki Hisamoto, Kunihiro Matsumoto

**Affiliations:** 1 Division of Biological Science, Graduate School of Science, Nagoya University, Chikusa-ku, Nagoya 464-8602, Japan; 2 Department of Biology, University of Utah, 257 South 1400 East, Salt Lake City, Utah 84112-0840, USA

## Abstract

The molecular mechanisms underlying the ability of axons to regenerate after injury remain poorly understood. Here we show that in *Caenorhabditis elegans*, axotomy induces ectopic expression of serotonin (5-HT) in axotomized non-serotonergic neurons via HIF-1, a hypoxia-inducible transcription factor, and that 5-HT subsequently promotes axon regeneration by autocrine signalling through the SER-7 5-HT receptor. Furthermore, we identify the *rhgf-1* and *rga-5* genes, encoding homologues of RhoGEF and RhoGAP, respectively, as regulators of axon regeneration. We demonstrate that one pathway initiated by SER-7 acts upstream of the *C. elegans* RhoA homolog RHO-1 in neuron regeneration, which functions via G12α and RHGF-1. In this pathway, RHO-1 inhibits diacylglycerol kinase, resulting in an increase in diacylglycerol. SER-7 also promotes axon regeneration by activating the cyclic AMP (cAMP) signalling pathway. Thus, HIF-1-mediated activation of 5-HT signalling promotes axon regeneration by activating both the RhoA and cAMP pathways.

## Abstract (web summary)

The molecular mechanism of axon regeneration after injury is poorly understood. Here the authors show that in *C. elegans*, the HIF-1 transcription factor induces ectopic synthesis of serotonin in severed ‘non-serotonergic' neurons and that serotonin activates downstream signaling pathways leading to axon regeneration.

## Graphical abstract

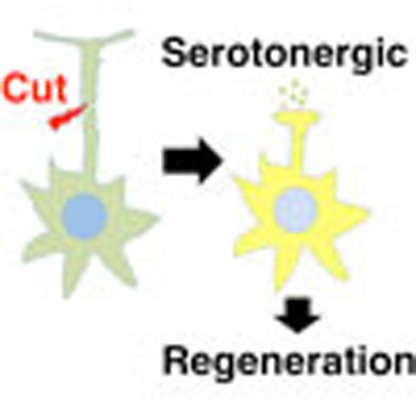

An axon's ability to regenerate after injury depends on both its intrinsic growth capacity and the external environment. Most invertebrate neurons, as well as neurons in the mammalian peripheral nervous system are able to regenerate. Although intrinsic regeneration signals can influence regenerative success, the variables determining a neuron's intrinsic regrowth capacity remain poorly defined at the molecular level. The nematode *Caenorhabditis elegans* has recently emerged as a tractable genetic model for studying neuron regenerative processes^1^,^2^. Two recent screens have identified candidate genes that may be involved in axon regeneration: Chen *et al.*^3^ screened a collection of mutants of *C. elegans*, and Nix *et al.*^4^ screened using an RNA interference (RNAi) library. The candidate genes identified include those encoding both growth-promoting and growth-inhibiting factors. Several of these genes have been implicated previously in regeneration and others define new and conserved pathways of interest.

In the present study, we investigate the roles of the *tph-1* and *rga-5* genes in the regulation of axon regeneration. The *tph-1* and *rga-5* genes were identified as candidate genes in a screen of mutants and by an RNAi screen, respectively^3^,^4^. The *tph-1* gene encodes an enzyme involved in 5-HT biosynthesis^5^, while the *rga-5* gene encodes a homolog of the mammalian p190 Rho GTPase-activating protein. We show that the hypoxia-inducible transcription factor (HIF-1) induces expression of the *tph-1* gene in axotomized neurons, which are not serotonergic. 5-HT subsequently promotes axon regeneration by signalling through the serotonin receptor SER-7. We further demonstrate that the RhoA signalling pathway is important for neuronal regeneration via its ability to inhibit diacylglycerol (DAG) kinase, thus increasing DAG concentrations. These two effects are linked, as *C. elegans* RhoA is activated by SER-7 and by G12α through RhoGEF encoded by the *rhgf-1* gene. SER-7 is also required for axon regeneration via its ability to activate the cyclic AMP (cAMP) signalling pathway. Thus, HIF-1-mediated activation of 5-HT synthesis and consequent autocrine signalling promotes the neuron regenerative response by activating two separate signalling pathways.

## Results

### 5-HT is required for axon regeneration

**Figure 1:**
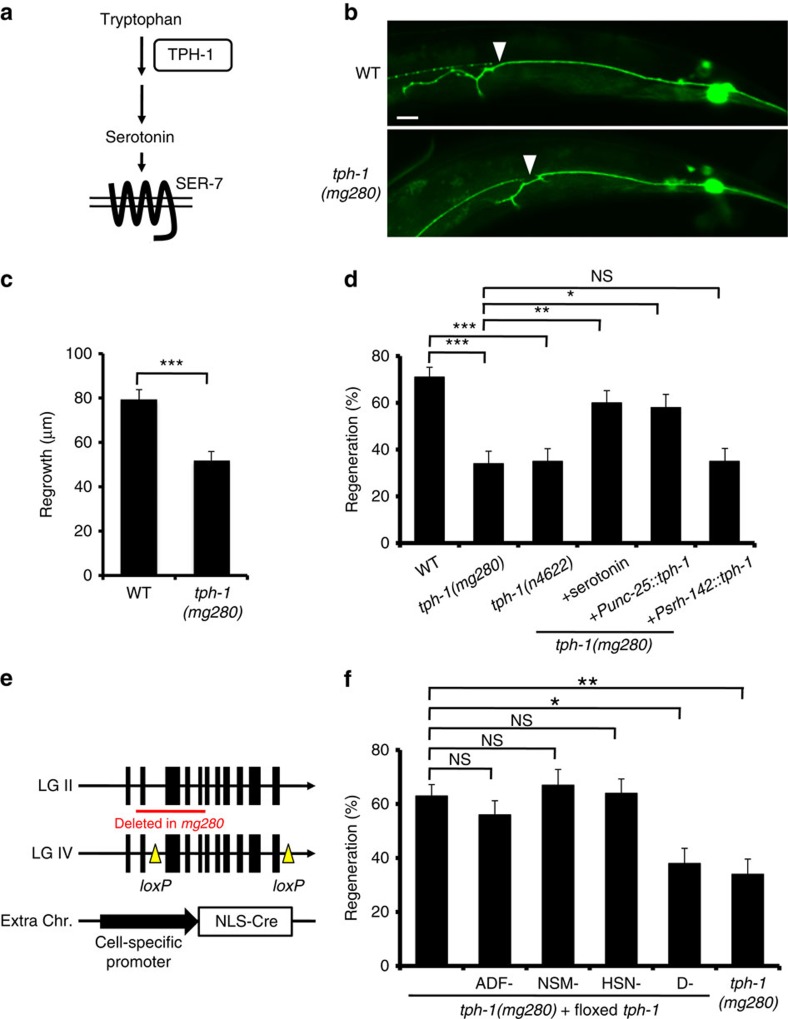
Serotonin activates axon regeneration. (**a**) The synthesis of serotonin from tryptophan catalyzed by TPH-1. (**b**) Representative PLM sensory neurons in wild type and *tph-1* mutant animals 24 h after laser surgery. Arrowheads indicate cut sites. Scale bar=20 μm. (**c**) Regeneration of PLM sensory neurons. Lengths of PLM regrowth at 24 h after laser surgery are shown. Error bars indicate s.e.m. ***P<0.001 (*n*≥30; unpaired *t*-test, two-tailed and unequal variances). (**d**,**f**) Regeneration of D-type motor neurons. Percentages of axons that initiated regeneration 24 h after laser surgery are shown. Error bars indicate 95% CI. ***P<0.001; **P<0.01; *P<0.05; NS, not significant (Fisher's exact test, two-tailed, *n*≥50). (**e**) Cell-specific deletion of *tph-1* using a Cre-*loxP* recombination system. CI, confidence interval.

It has been suggested that axon injury may represent a type of cellular stress. In response to specific types of stress, neurotransmitters are deployed to effect the appropriate physiological and behavioural modifications^6^,^7^,^8^. In *C. elegans*, 5-HT modulates a variety of stress responses^9^,^10^. However, little is known about how specific types of stress induce 5-HT-mediated signal transduction in the nervous system. Interestingly, Chen *et al.*^3^ recently carried out a systematic screen of mutants looking for defects in axon regeneration and identified *tph-1* among several genes that positively regulate axon regeneration in a glutaminergic touch sensory neuron^3^. The *tph-1* gene encodes tryptophan hydroxylase^5^, which is required for 5-HT biosynthesis (Fig. 1a). We confirmed that a loss-of-function mutation (*mg280*) of *tph-1* caused a defect in axon regeneration in touch sensory posterior lateral microtubule (PLM) neurons (Fig. 1b,c and Supplementary Table 1). Furthermore, the m*g280* and *n4622* mutations of *tph-1* were also defective in axon regeneration in GABA-releasing D-type motor neurons (Fig. 1d and Supplementary Table 2). Exogenous 5-HT treatment restored axon regeneration in *tph-1* mutants. These results suggest that 5-HT is generally required during axon regeneration.

We next searched for the 5-HT-producing neurons essential for axon regeneration. To match serotonin levels to the normal range for each neuron, we used a system recently developed by Flavell *et al.*^11^ A single floxed copy of the *tph-1* gene, including the promoter, exons and introns, was inserted into a defined location on chromosome IV (Fig. 1e). This inserted gene was able to rescue the *tph-1* defect in axon regeneration of D-type motor neurons (Fig. 1f and Supplementary Table 2). This was combined with cell-specific transgenes expressing Cre recombinase in amphid sensory neuron, dual cilia, designation F. (ADF), neurosecretory motor neuron (NSM) or hermaphrodite specific neuron (HSN) neurons, which are known to be 5-HT-producing neurons^5^, or in D-type neurons. This system allowed the excision of the inserted *tph-1* gene and elimination of 5-HT production in these individual neurons. We found that the *tph-1* defect was still rescued when Cre was expressed in ADF, NSM or HSN neurons (Fig. 1f and Supplementary Table 2). In contrast, the *tph-1* defect was not rescued when Cre was expressed in D-type motor neurons. Furthermore, expression of *tph-1* in ADF neurons by the *srh-142* promoter was unable to rescue the *tph-1* defect (Fig. 1d and Supplementary Table 2). These results suggest that axon regeneration in D-type motor neurons requires 5-HT production by D neurons. To confirm this, we ectopically expressed *tph-1* in D-type motor neurons in a *tph-1(mg280)* background using the *unc-25* promoter. We found that expression of *tph-1* in D neurons did indeed suppress the defect in axon regeneration of *tph-1* mutants (Fig. 1d and Supplementary Table 2). These results demonstrate that TPH-1 acts to promote regeneration of the damaged neuron in a cell-autonomous manner.

### HIF-1 upregulates 5-HT expression in injured neurons

**Figure 2:**
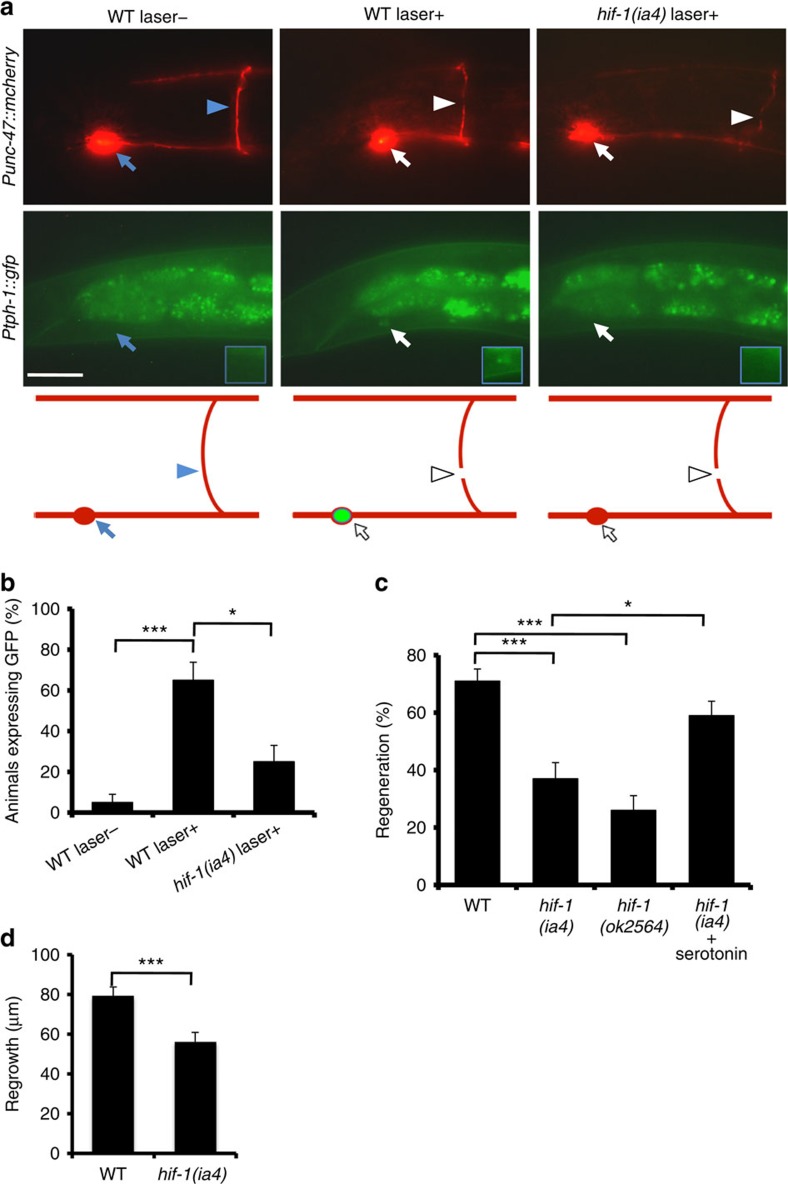
HIF-1 positively regulates axon regeneration. (**a**) Induction of *Ptph-1::gfp* expression in D-type motor neurons by laser surgery. Expression of fluorescent proteins in D-type motor neurons 30 min after laser surgery is shown. A schematic representation of D-type motor neurons is shown on the bottom. Arrowheads and arrows indicate axons and cell bodies of D-type neurons with (white) or without (blue) laser surgery, respectively. D-type neurons are visualized by mCherry under control of the *unc-47* promoter. Cell bodies of D-type neurons are magnified (blue boxes). Scale bar=20 μm. (**b**) Percentages of animals expressing *tph-1*. Induction of *Ptph-1::gfp* expression in D-type motor neurons with (+) or without (−) laser surgery was assayed as described in Methods section. Twenty neurons were examined for each condition. Error bars indicate 95% CI. ***P<0.001; *P<0.05 (Fisher's exact test, two-tailed, *n*=20). (**c**) Regeneration of D-type motor neurons. Percentages of axons that initiated regeneration 24 h after laser surgery are shown. Error bars indicate 95% CI. ***P<0.001; *P<0.05 (Fisher's exact test, two-tailed, *n*≥50). (**d**) Regeneration of PLM sensory motor neurons. Lengths of PLM regrowth at 24 h after laser surgery are shown. Error bars indicate s.e.m. ***P<0.001 (*n*≥30; unpaired *t*-test, two-tailed, unequal variances). CI, confidence interval.

The identification of D-type neurons as the 5-HT-producing neurons essential for axon regeneration was unexpected, as D neurons are known to be GABAergic and the expression of 5-HT synthesis enzymes in these neurons has not been found^5^,^12^,^13^,^14^. We therefore addressed the possibility that axon severing might induce transcription of the *tph-1* gene in D-type neurons. To test this, we monitored expression of the *Ptph-1::gfp* transgene, which consists of the *tph-1* promoter driving the fluorescent protein green fluorescent protein (GFP). We found that expression of *tph-1* was altered in D-type neurons by axon injury. In control animals, *Ptph-1::gfp* was robustly expressed in the head of animals in the NSM and ADF neurons (Supplementary Fig. 1), and was not expressed in D neurons. However, after laser surgery of D-type neurons, *Ptph-1::gfp* expression was strongly upregulated in these neurons (Fig. 2a,b). This expression became visible at 30 min after surgery, and returned to undetectable levels by 24 h. Thus, axon injury induces the transient expression of *Ptph-1::gfp* in severed D-type neurons. 5-HT immunofluorescence staining of animals treated with laser surgery also revealed induction of 5-HT expression, compared with control animals (Supplementary Fig. 2). Expression of *Punc-47::mcherry*, which reflects the expression of vesicular GABA transporter, was constant over this time period (Fig. 2a). Therefore, the severed GABAergic neurons still keep the GABAergic characteristics, and do not transdifferentiate into serotonergic, non-GABAergic neurons. Furthermore, axon severing also induced 5-HT and *Ptph-1::gfp* expression in PLM neurons (Supplementary Figs 2 and 3), which are glutamatergic, non-serotonergic touch sensory neurons^15^. Our results demonstrate that axotomized ‘non-serotonergic' neurons temporarily gain the ability to synthesize serotonin in response to axon injury.

What determines expression of the ectopic *tph-1* gene in response to axon injury? Interestingly, the promoter region of the *tph-1* gene contains hypoxia response elements, which are binding sites for HIF-1 (ref. 16). The HIF-1 protein is a transcription factor containing a bHLH-PAS domain that mediates transcriptional responses to hypoxic stress^17^. Indeed, it has been demonstrated that HIF-1 binds to the *tph-1* promoter via hypoxia response elements to drive serotonin production under hypoxic conditions in *C. elegans*^16^. We therefore examined whether HIF-1 is involved in *tph-1* expression in response to axon injury. The *hif-1(ia4)* mutation had no effect on expression of *Ptph-1::gfp* in the head of animals (Supplementary Fig. 1) but decreased its induction in response to laser surgery in D-type neurons (Fig. 2a,b), indicating that induction is dependent on HIF-1 function. In addition, expression of *Ptph-1::gfp* in PLM neurons following laser surgery decreased in *hif-1* mutants (Supplementary Fig. 3). Thus, HIF-1 is required for induction of 5-HT expression in injured neurons in response to axon injury.

These results raised the possibility that hypoxic stress might enhance axon regeneration. In normoxic conditions, HIF-1 is degraded through the E3 ubiquitin ligase VHL-1 (von Hippel–Lindau tumour suppressor protein)-dependent degradation pathway^18^. Degradation is initiated through hydroxylation of a specific proline residue in HIF-1 by the oxygen-dependent prolyl 4-hydroxylase EGL-9 (ref. 18). VHL-1 recognizes the hydroxylated proline and targets HIF-1 for ubiquitin-mediated proteasomal degradation^19^. Hypoxic conditions inhibit the hydroxylation of HIF-1, stabilizing the protein and allowing it to transcriptionally regulate expression of its target genes^19^,^20^. Thus, stabilization of HIF-1 through removal of the HIF-1 degradatory pathway phenocopies the effects of hypoxia seen in neurons. We examined the effect of HIF-1 stabilization on *tph-1* expression in D-type motor neurons. For this purpose, we used *vhl-1(ok161)* mutants and a mutated form of HIF-1, HIF-1(P621A), in which the proline in the conserved LXXLAP motif required for degradation of HIF-1 has been altered to an alanine^18^,^21^. As observed previously^16^, the *vhl-1* mutation or expression of HIF-1(P621A) caused constitutive expression of *tph-1* in glutamatergic sensory amphid sensory neuron, single cilia, designation G. (ASG) neurons under normoxic conditions (Supplementary Figs 1 and 4). Normally, *tph-1* is only weakly expressed under normoxic conditions in these neurons. However, we found that the *vhl-1* mutation or expression of HIF-1(P621A) failed to induce *Ptph-1::gfp* expression constitutively in D neurons (Supplementary Fig. 4). These results suggest that the mechanism that activates HIF-1 in response to axon injury is different from the HIF-1-stabilizing mechanism that is involved in the response to hypoxia.

Next, we examined whether HIF-1 is required for 5-HT-driven axon regeneration. We found that the frequency of axon regeneration in D neurons was reduced significantly, in *hif-1(ia4)* and *hif-1(ok2564)* mutants, and was restored by exogenous 5-HT treatment (Fig. 2c and Supplementary Table 2). This is consistent with the possibility that HIF-1 regulates axon regeneration by driving 5-HT production. Furthermore, the *hif-1(ia4)* mutation was also defective in axon regeneration in PLM neurons following laser surgery (Fig. 2d and Supplementary Table 1). These results suggest that HIF-1 is generally required during axon regeneration.

### SER-7 activates axon regeneration via GPA-12 and RHGF-1

**Figure 3:**
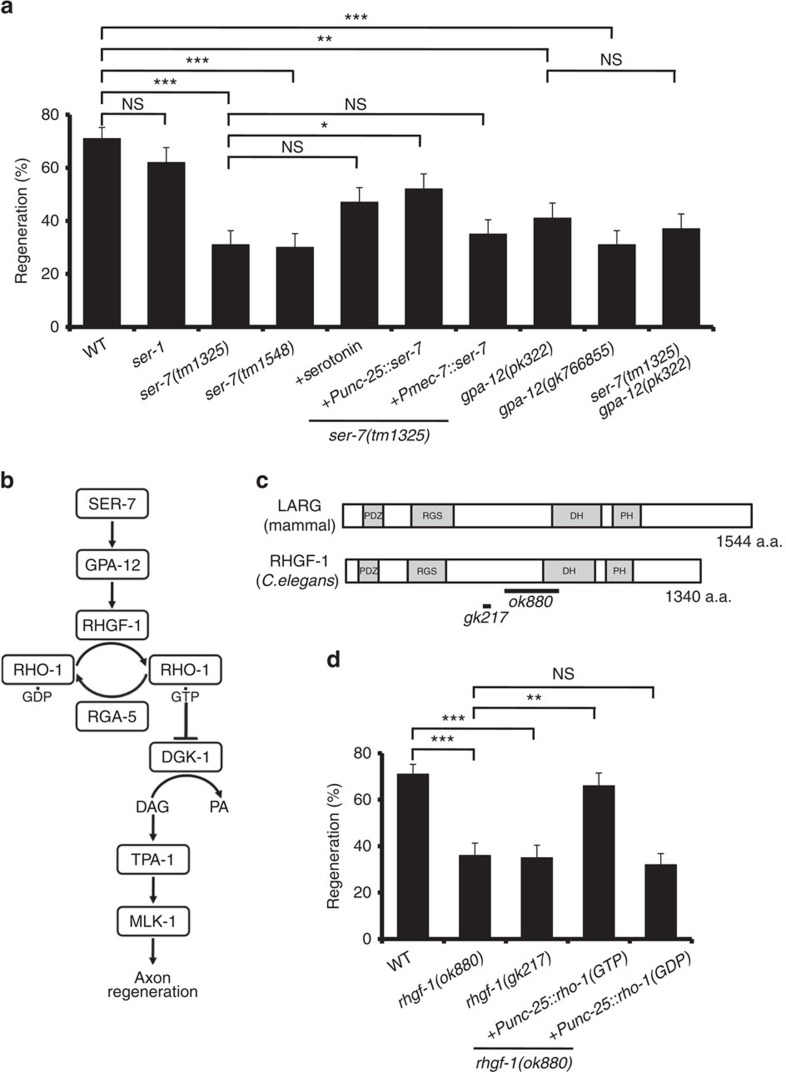
5-HT-SER-7 signalling activates axon regeneration via GPA-12 and RHGF-1. (**a**,**d**) Percentages of axons that initiated regeneration 24 h after laser surgery. Error bars indicate 95% CI. ***P<0.001; **P<0.01; *P<0.05; NS, not significant (Fisher's exact test, two-tailed, *n*≥50). (**b**) SER-7 signalling pathway. SER-7 activates the GPA-12-RHGF-1-RHO-1 pathway. Activation of RHO-1 inhibits DGK-1, leading to a stabilization of DAG levels. (**c**) Structure of RHGF-1. Schematic diagrams of RHGF-1 and its human counterpart are shown. Domains are shown as follows: a PDZ domain, a regulator of G protein signalling domain (RGS), a Dbl homology domain (DH) and a Pleckstrin homology domain (PH). The bold lines underneath indicate the extent of the deleted regions in the *rhgf-1(ok880)* and *rhgf-1(gk217)* mutants.

The biological effects of 5-HT are mediated through its interactions with G protein-coupled receptors^22^. Since SER-7 is one of the 5-HT receptors, we examined whether SER-7 participates in axon regeneration. We found that two mutations (*tm1325* and *tm1548*) in the *ser-7* gene caused defects in axon regeneration (Fig. 3a and Supplementary Table 2). In contrast to *ser-7*, the *ok345* null mutation of *ser-1* encoding another serotonin receptor had no effect on axon regeneration. Thus, SER-7 plays a specific role in axon regeneration. To test whether SER-7 can act in a cell-autonomous manner, we expressed *ser-7* from the *unc-25* or *mec-7* promoters in *ser-7(tm1325)* mutants. The *ser-7* defect in axon regeneration was rescued by expression of *ser-7* in D-type motor neurons by the *unc-25* promoter but not by expression in sensory neurons by the *mec-7* promoter (Fig. 3a and Supplementary Table 2). These results demonstrate that SER-7 acts to promote regeneration in a cell-autonomous manner in the damaged neuron. Exogenous 5-HT treatment was unable to efficiently suppress the *ser-7* defect (Fig. 3a and Supplementary Table 2).

What functions downstream of SER-7 to regulate axon regeneration? SER-7 is coupled to GPA-12, a *C. elegans* homologue of the α subunit of the heterotrimeric G12 protein (Fig. 3b)^23^. We found that the *gpa-12(pk322)* and *gpa-12(gk766855)* mutations significantly reduced axon regeneration after axon injury (Fig. 3a and Supplementary Table 2). To further address whether *ser-7* and *gpa-12* function in the same pathway, we constructed *ser-7(tm1325)*; *gpa-12(pk322)* double mutants. We found that the *gpa-12* mutation did not enhance the axon regeneration defect of *ser-7* mutants (Fig. 3a and Supplementary Table 2), suggesting that SER-7 and GPA-12 function in the same pathway.

In mammals, the RhoGEF LARG protein specifically interacts with G12α via its regulator of G protein signalling (RGS) domain^24^. *C. elegans* RHGF-1, a homolog of mammalian LARG (Fig. 3c), binds to GPA-12 and acts as a regulator of neurotransmitter release at a subset of cholinergic motor synapses^25^,^26^. These observations raised the possibility that RHGF-1 may act downstream of GPA-12 in the axon regeneration pathway (Fig. 3b). Indeed, we found that the *rhgf-1(ok880)* and *rhgf-1(gk217)* mutants were defective in axon regeneration (Fig. 3d and Supplementary Table 2). Taken together, these results suggest that SER-7 activates axon regeneration through GPA-12 and RHGF-1.

### RHO-1 activates axon regeneration by inhibiting DGK-1

The activity of Rho proteins is modulated by their cycling between the active GTP- and inactive GDP-bound states. Rho activity is further regulated by other proteins, including GAPs, which enhance GTP hydrolysis and the consequent inactivation of Rho, and guanine nucleotide exchange factors (GEFs), which promote the binding of GTP and, thus, activation of Rho^27^. RHO-1 also cycles between the GTP-bound active and the GDP-bound inactive forms. To confirm that RHGF-1 acts as a GEF for RHO-1 and to test if it thereby controls axon regeneration, we expressed a mutant RHO-1 that is locked in the GTP-bound state, RHO-1(G14V), in D-type motor neurons using the *unc-25* promoter. We found that expression of RHO-1(G14V) was able to suppress the *rhgf-1* defect in axon regeneration, whereas a GDP-bound RHO-1(T19N) expressed from the *unc-25* promoter was not (Fig. 3d and Supplementary Table 2).

**Figure 4:**
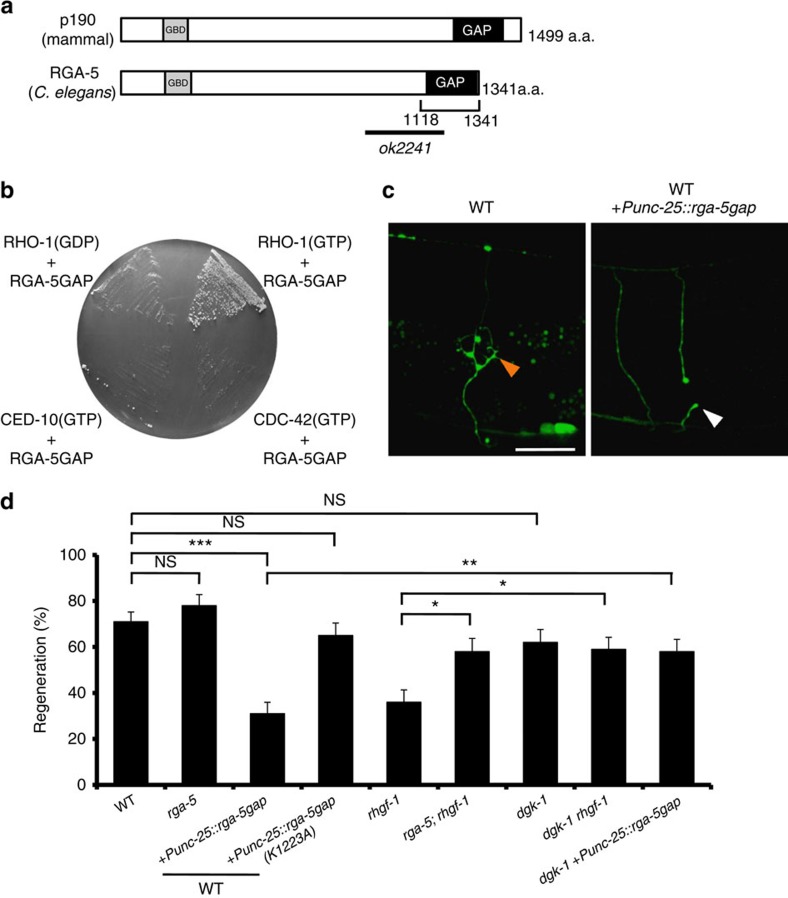
Effect of RhoGAP on axon regeneration. (**a**) Structure of RGA-5. Schematic diagrams of RGA-5 and its human counterpart are shown. Domains are shown as follows: a GTP binding domain (GBD) and a Rho GAP domain (GAP). The bold line underneath indicates the extent of the deleted region in the *rga-5(ok2241)* mutant. (**b**) A two-hybrid assay for the interaction of RGA-5 with RHO-1. The reporter strain PJ69-4A was co-transformed with expression vectors encoding GAL4 DBD-RHO-1 (T19N) (GDP), GAL4 DBD-RHO-1 (G14V) (GTP), GAL4 DBD-CED-10 (G12V) (GTP), GAL4 DBD-CDC-42 (G12V) (GTP) and GAL4 AD-RGA-5 (GAP domain) as indicated. (**c**) Representative D-type motor neurons in wild-type animals and wild-type animals overexpressing RGA-5 GAP domain 24 h after laser surgery. In wild-type animals, severed axons have regenerated growth cones (orange arrowhead). In wild-type animals overexpressing the RGA-5 GAP domain, the proximal end of axon failed to regenerate (white arrowhead). Scale bar=20 μm. (**d**) Percentages of axons that initiated regeneration 24 h after laser surgery. Error bars indicate 95% CI. ***P<0.001; **P<0.01; *P<0.05; NS, not significant (Fisher's exact test, two-tailed, *n*≥50). CI, confidence interval.

What functions as a RhoGAP in the regulation of axon regeneration? One of the genes identified in our previous RNAi screen in β-spectrin mutant animals (*unc-70* ; ref. 28) was *rga-5*, the product of which belongs to the Rho GTPase-activating protein (GAP) family^4^. RGA-5 has an N-terminal GTP binding domain (GBD) and a C-terminal GAP domain. RGA-5 displays strong homology across its GBD and GAP domains to the mammalian p190 RhoGAP (Fig. 4a and Supplementary Fig. 5). Since the GAP domain in p190 RhoGAP is specific for the Rho family of GTPases, particularly RhoA (ref. 29), we expected that RGA-5 would specifically act on RHO-1. To test this prediction, we performed yeast two-hybrid binding assays to test for interactions between RGA-5 and two RHO-1 variants that are locked in the GTP- and GDP-bound states (G14V and T19N, respectively). We found that the RGA-5 GAP domain (1118–1341 amino acids) specifically interacted with the GTP bound, but not GDP-bound version of RHO-1 (Fig. 4b), consistent with the property of GAP proteins to associate with the GTP-bound version of GTPases. The RGA-5 GAP domain failed to bind to GTP-bound CDC-42 or CED-10, two other Rho/Rac family members, demonstrating that the RGA-5-RHO-1 interaction is specific.

Next, we determined the function of RGA-5 in axon regeneration. First, we examined the *rga-5*(*ok2241*) mutation, which deletes the GAP domain in RGA-5 (Fig. 4a). After laser surgery, axons in *rga-5*(*ok2241*) mutant animals regenerated normally. However, overexpression of the RGA-5 GAP domain reduced regeneration (Fig. 4c,d and Supplementary Table 2). It has been confirmed that the GAP domain of p190 RhoGAP possesses GAP activity and that the conserved Lys-1311 (Lys-1223 in RGA-5) residue is essential for this activity (Supplementary Fig. 5b)^29^,^30^. Overexpression of the mutant GAP(K1223A), in which the Lys-1223 in the RGA-5 GAP domain was mutated to alanine, did not cause reduced regeneration (Fig. 4d and Supplementary Table 2). These results suggest that the RGA-5 GAP domain stimulates the conversion of RHO-1 to the GDP-bound inactive form, resulting in the inhibition of axon regeneration. In this manner, RGA-5 acts as a potent inhibitor of axon regeneration. We further examined possible genetic interactions between *rga-5* and *rhgf-1*, and found that the *rga-5(ok2241)* mutation was able to suppress the *rhgf-1(ok880)* defect (Fig. 4d and Supplementary Table 2). This can be explained because RHO-1 can still be weakly activated in the absence of its GEF, thus concomitant loss of GAP affects the phenotype.

How does RHO-1 regulate axon regeneration? Mammalian RhoA has been shown to bind and inhibit the DAG kinase DGK*θ* (refs 31, 32). DAG kinases remove DAG by phosphorylating the membrane-bound secondary messenger DAG, converting it to phosphatidic acid^33^,^34^, which implicates RhoA in regulating DAG levels^31^. In addition, *C. elegans* RHO-1 is able to bind to DGK-1, the *C. elegans* ortholog of DGK*θ* (ref. 35). Thus, DGK-1 is a possible target of negative regulation by RHO-1 (Fig. 3b). If RHO-1 modulates axon regeneration through the inhibition of DGK-1, then we would predict that a *dgk-1* loss-of-function mutation should suppress the regeneration defect by inactivation of RHO-1. As expected, we found that the *dgk-1(ok1462)* null mutation suppressed the defect caused by the *rhgf-1(ok880)* deletion mutation or overexpression of the RGA-5 GAP domain (Fig. 4d and Supplementary Table 2). Our results are most consistent with a signal transduction cascade involving activation of RHO-1, inhibition of DGK-1, and a consequent increase in DAG (Fig. 3b).

### SER-7 promotes axon regeneration via the cAMP pathway

**Figure 5:**
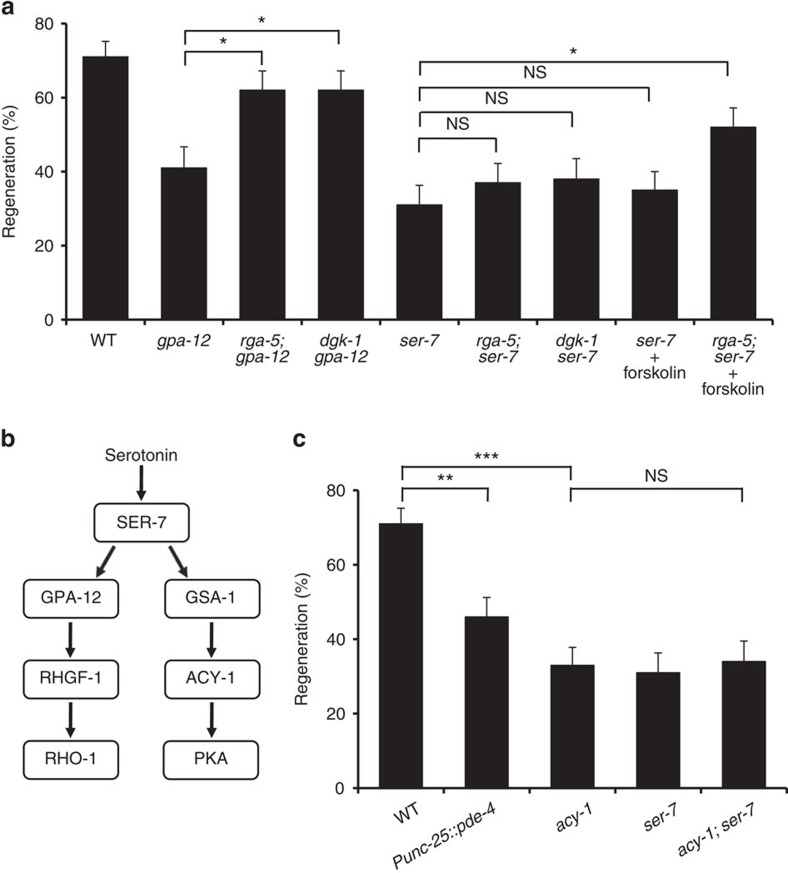
SER-7 promotes axon regeneration via the cAMP pathway. (**a**,**c**) Percentages of axons that initiated regeneration 24 h after laser surgery. Error bars indicate 95% CI. ***P<0.001; **P<0.01; *P<0.05; NS, not significant (Fisher's exact test, two-tailed, *n*≥50). (**b**) SER-7 signalling pathways. SER-7 activates both GPA-12-RHGF-1-RHO-1 and GSA-1-ACY-1-PKA pathways.

If the SER-7-GPA-12 pathway acts upstream of RHO-1 to promote axon regeneration, an *rga-5* or *dgk-1* mutation should suppress the defect in axon regeneration seen in *ser-7* and *gpa-12* mutants. As expected, the *rga-5(ok2241)* and *dgk-1(ok1462)* mutations were each able to suppress the *gpa-12* defect (Fig. 5a and Supplementary Table 2). However, in contrast to the *gpa-12* mutation, the *ser-7* defect was not suppressed by the *rga-5(ok2241)* or *dgk-1(ok1462)* mutation (Fig. 5a and Supplementary Table 2). These results suggest that another regulatory pathway exists downstream of SER-7, in addition to the GPA-12-RHO-1 pathway, that is required for axon regeneration.

Since the SER-7 5-HT receptor has also been reported to be coupled to GSA-1 Gsα (Fig. 5b)^23^,^36^, we hypothesized that an additional pathway of SER-7-mediated axon regeneration could involve the GSA-1 signalling pathway. Since Gsα is coupled to adenylyl cyclase, Gsα signalling can be monitored by intracellular cAMP levels. As observed previously^37^, axon regeneration was inhibited by overexpression of *pde-4*, which encodes a cAMP-phosphodiesterase and reduces intracellular cAMP levels (Fig. 5c and Supplementary Table 2). Furthermore, a loss-of-function mutation of *acy-1*, which encodes adenylyl cyclase, was defective in axon regeneration (Fig. 5c and Supplementary Table 2). These results suggest that activation of GSA-1 signalling is necessary to activate axon regeneration. To confirm that *ser-7* and *acy-1* function in the same pathway, we constructed *ser-7(tm1325)*; *acy-1(nu329)* double mutants. The *ser-7* mutation did not enhance the axon regeneration defect of *acy-1* mutants (Fig. 5c and Supplementary Table 2), supporting the possibility that SER-7 functions in the GSA-1 signalling pathway to activate axon regeneration.

Since SER-7 activates both GPA-12 and GSA-1 signalling pathways (Fig. 5b)^23^, we speculated that activation of both pathways are required for axon regeneration. If so, activation of the GSA-1 signalling pathway should suppress the *ser-7* defect in axon regeneration in a manner dependent on the activation of the GPA-12 signalling pathway. Treatment of animals with forskolin is expected to cause an increase in cAMP levels by activating adenylyl cyclase^37^,^38^. We found that forskolin treatment failed to suppress the single mutant *ser-7(tm1325)* defect in axon regeneration (Fig. 5a and Supplementary Table 2). However, forskolin treatment was able to suppress the axon regeneration defect in *ser-7*; *rga-5* double mutants, in which the GPA-12 signalling pathway is activated (Fig. 5a and Supplementary Table 2). These results suggest that SER-7 activates GPA-12 and GSA-1, which act in concert to activate axon regeneration.

## Discussion

The neurotransmitter 5-HT plays multiple roles in the enteric, peripheral and central nervous system, and its most prominent biological function is as a messenger transmitting signals from pre- to post-synaptic neurons. However, 5-HT has been reported to play other roles, such as the regulation of neurite growth^39^,^40^, suggesting that 5-HT also plays a prominent role in regulating the neuronal cytoarchitecture^41^,^42^. In *C. elegans*, 5-HT modulates cognitive behaviours such as chemosensation, learning and memory in addition to a variety of stress responses^10^,^13^,^16^. Here our results demonstrate that *C. elegans* 5-HT is synthesized in axotomized ‘non-serotonergic' neurons and is required for axon regeneration. This effect is likely due to a direct action of 5-HT on injured neurons through the SER-7 5-HT receptor, which activates axon regeneration in a cell-autonomous manner.

Neurons are known to be able to produce distinct sets of neurotransmitters^43^, and some studies have shown that certain stimuli can induce neurotransmitter switching^44^,^45^. In mammals, leukaemia inhibitory factor and ciliary neurotrophic factor have been shown to induce the differentiation of developing peripheral sympathetic neurons, which normally release noradrenaline, into cholinergic neurons^46^,^47^. Another study revealed that the balance of the two hippocampal granule cell neurotransmitters, glutamate and GABA, changes during post-embryonic development, as well as with seizures and long-term potentiation^48^. Recently, Dulcis *et al.*^49^ reported that the neurotransmitters produced by hypothalamic neurons switch between dopamine and somatostatin in response to photoperiod stimuli. Although these studies were largely carried out using populations of neuronal cells that could include different types of cells such as reserve pool neurons, they indicate that neurotransmitter production varies dynamically in neurons. Our findings in *C. elegans* demonstrate that a single, differentiated GABAergic D-type motor neuron or glutamatergic PLM sensory neuron that is never serotonergic under standard laboratory conditions can be induced to produce 5-HT as a result of axotomy in a manner dependent on HIF-1. This represents a novel case in which a switch in neurotransmitter expression can be regulated. Hypoxia also stimulates expression of 5-HT via HIF-1 in glutamatergic ASG neurons^16^,^50^. However, stabilization of HIF-1, which phenocopies the hypoxia-induced neuronal effects, is sufficient to induce *tph-1* expression in ASG neurons, but not in D or PLM neurons. Therefore, HIF-1-dependent *tph-1* expression in severed neurons is regulated by a mechanism different from the HIF-1-stabilizing mechanism.

In mammals, the expression and stability of HIF-1, as well as the expression of its target genes, are all increased after spinal cord injury^51^,^52^. Thus, activation of HIF-1 by axotomy may be conserved among animals. Furthermore, the HIF-induced upregulation of 5-HT in specific regions of the vertebrate brain^53^ suggests that the mechanisms coupling axon regeneration and 5-HT signalling to produce an alteration in neuronal responses may be conserved among animals. Therefore, it would be interesting to know whether axotomy of vertebrate neurons also stimulates expression of 5-HT or other neurotransmitters that influence regeneration. The inability of axons to regenerate in the adult mammalian central nervous system is a major problem in a number of diseases, and understanding the multi-functional roles of 5-HT signalling on neurite growth may lead to the development of novel neuroregenerative therapies by targeting serotonin-regulated signalling pathways.

The biological effects of 5-HT are mediated through interactions with G protein-coupled receptors^22^. Here we have shown that the activated SER-7 5-HT receptor promotes axon regeneration in injured neurons by activating G12α and Gsα signalling pathways in a cell-autonomous manner. How do G12α and Gsα signalling cascades activate axon regeneration? Several studies suggested that DAG and cAMP act downstream of the G12α and Gsα cascades, respectively^54^,^55^,^56^,^57^. In mammals, G12α activates an RGS-containing RhoGEF, which in turn activates RhoA. Several lines of evidence suggest that this pathway also acts within *C. elegans* motor neurons^26^. The axon regeneration pathway is regulated by both of these components acting in the G12α pathway, as either a mutation in *rhgf-1* encoding the RGS-containing RhoGEF or inactivation of RHO-1 by RhoGAP RGA-5 inhibits axon regeneration. In addition, RHO-1 activation is able to suppress the defect in axon regeneration caused by the *gpa-12* mutation. Thus, the genetic data support a model in which the G12α-RhoGEF-RhoA signalling pathway is conserved in the control of axon regeneration in *C. elegans*. Consistent with this, GPA-12 and RHGF-1 are both expressed in motor neurons and physically interact^25^. In *C. elegans*, activated RHO-1 causes an increase in DAG by directly binding to and inhibiting DGK-1 (ref. 35). High levels of DAG activate the PKC homolog TPA-1, leading to activation of the JNK pathway by phosphorylation of MLK-1 MAPKKK^58^. These results raise the possibility that the G12α pathway activates axon regeneration by inducing an increase in DAG, resulting in the activation of the JNK pathway.

The Gsα cascade involves the activation of adenylyl cyclase by Gsα, and the consequent increase in cAMP production leads to the activation of PKA. Our recent results suggest that in response to axon injury, PKA upregulates transcription of the *svh-2* gene, which encodes a growth factor receptor functioning in the JNK pathway^59^,^60^. Thus, one effector of the Gsα pathway that stimulates axon regeneration is a transcription factor that activates transcription of the *svh-2* gene, resulting in activation of the JNK pathway. However, the Gsα pathway likely has additional targets. Taken together, these results suggest that the G12α and Gsα pathways converge to activate the JNK pathway in axon regeneration.

## Methods

### *C. elegans* strains

*C. elegans* strains used in this study are listed in Supplementary Table 3. All strains were maintained on nematode growth medium (NGM) plates and fed with bacteria of the OP50 strain, as described previously^61^.

### Plasmids

*Punc-25::tph-1* was made by inserting the *tph-1* cDNA isolated from a cDNA library by PCR into the pSC325 vector. *Psrh-142::tph-1* was made by replacing an NLS-Cre cDNA fragment of *Psrh-142::nls-cre* plasmid^11^ (Gift from Dr Cori Bargmann) with a *tph-1* cDNA fragment. *Punc-25::nls-cre* was generated by inserting NLS-Cre cDNA (Addgene) into the pSC325 vector. *Punc-47::mcherry* was generated by replacing the *gfp* DNA of the pOKU64 vector (*Punc-47::gfp*, a gift from Dr Ikue Mori) with the mCherry cDNA (DNA2.0). *Punc-25::ser-7* and *Pmec-7::ser-7* were made by inserting the *ser-7* cDNA isolated from a cDNA library by PCR into the pSC325 and pPD52.102 vectors, respectively. *Punc-25::rho-1* was generated by inserting the *rho-1* cDNA, provided from Dr Kozo Kaibuchi (Nagoya University, JAPAN), into the pSC325 vector. *Punc-25::rho-1 (G14V)* and *Punc-25::rho-1 (T19N)* were made by oligonucleotide-directed PCR using *Punc-25::rho-1* as a template and the mutations were verified by DNA sequencing. The *Punc-25::rga-5gap* plasmid contains the GAP domain of *rga-5* cDNA isolated from a cDNA library by PCR using oligonucleotides 5′-gtcagcATGAAGAACAAAGTGCAACTCATGATC-3′ and 5′-ccatGGCCCGATAATTATCCACGTCATAG-3′, inserted into the pSC325 vector. The *Punc-25::rga-5gap(K1223A)* was made by oligonucleotide-directed PCR using *Punc-25::rga-5gap* as a template and the mutation was verified by DNA sequencing. *Punc-25::pde-4* was generated by inserting the *pde-4* cDNA isolated from a cDNA library into the pSC325 vector. The GAL4 DBD-RHO-1 (G14V), GAL4 DBD-RHO-1 (T19N), GAL4 DBD-CED-10 (G12V) and GAL4 DBD-CDC-42 (G12V) plasmids are also generous gifts from Dr Kozo Kaibuchi. The GAL4 AD-RGA-5 (GAP domain) plasmid was constructed by inserting the *rga-5gap* cDNA into the pACTII vector. The *Pmyo-2::dsred-monomer* plasmid has been described previously^59^.

### Transgenic animals

Transgenic animals were obtained as described in ref. 62. *Punc-25::rga-5gap* (50 ng μl^−1^), *Punc-25::rga-5gap(K1223A)* (50 ng μl^−1^), *Punc-25::rho-1 (G14V)* (5 ng μl^−1^), *Punc-25::rho-1 (T19N)* (5 ng μl^−1^), *Punc-25::ser-7* (50 ng μl^−1^), *Pmec-7::ser-7* (5 ng μl^−1^), *Punc-25::pde-4* (50 ng μl^−1^), *Punc-25::tph-1* (50 ng μl^−1^), *Psrh-142::tph-1* (50 ng μl^−1^), *Punc-25::nls-cre* (50 ng μl^−1^) and *Punc-47::mcherry* (100 ng μl^−1^) plasmids were used to generate *kmEx1301* [*Punc-25::rga-5gap*+*Pmyo-2::dsred-monomer*], *kmEx1312* [*Punc-25::rga-5gap(K1223A)*+*Pmyo-2::dsred-monomer*], *kmEx1302* [*Punc-25::rho-1 (G14V)*+*Pmyo-2::dsred-monomer*], *kmEx1303* [*Punc-25::rho-1 (T19N)*+*Pmyo-2::dsred-monomer*], *kmEx1304* [*Punc-25::ser-7*+*Pmyo-2::dsred-monomer*], *kmEx1305* [*Pmec-7::ser-7*+*Pmyo-2::dsred-monomer*], *kmEx1306* [*Punc-25::pde-4*+*Pmyo-2::dsred-monomer*], *kmEx1307* [*Punc-25::tph-1*+*Pmyo-2::dsred-monomer*], *kmEx1311* [*Psrh-142::tph-1*+*Pmyo-2::dsred-monomer*], *kmEx1308* [*Punc-25::nls-cre*+*Pmyo-2::dsred-monomer*] and *kmEx1309* [*Punc-47::mcherry*+*Pmyo-2::dsred-monomer*], respectively. The *kyEx4057* [*Pceh-2::nls-cre*], *kyEx4077* [*Psrh-142::nls-cre*], *kyEx4107* [*Pegl-6::nls-cre*] and *kySi56* [floxed *tph-1* genomic rescue] were generous gifts from Dr Cori Bargmann^11^. The *otIs197*, *zdIs13*, *wpIs36* and *wdEx848* transgenes^63^,^64^,^65^ were obtained from CGC.

### Yeast two-hybrid assay

GAL4 DBD-RHO-1(G14V, T19N), GAL4 DBD-CED-10 (G12V), GAL4 DBD-CDC-42 (G12V) and GAL4 AD-RGA-5 (GAP domain) plasmids were co-transformed into the *Saccharomyces cerevisiae* reporter strain PJ69-4A and allowed to grow on SC-Leu-Trp plates. Transformants grown on these plates were then streaked out onto SC-Leu-Trp-His plates and incubated at 30 °C for 4 days.

### Axotomy

All animals were subjected to axotomy at the young adult stage at 20 °C. Young adult hermaphrodites were immobilized with 20 mM sodium azide in M9 buffer on 2% agarose pad under a cover slip. D-type motor neurons expressing GFP were imaged with a fluorescence microscope. Selected commissural axons, mainly posterior D-type neurons, were cut using a 440-nm MicroPoint ablation Laser System from Photonic Instruments. After surgery, animals were allowed to recover on an agar plate and remounted for fluorescent imaging ∼24 h after surgery. For measurements of D neurons, imaged commissures that exhibited growth cones or small branches on the proximal fragment were counted as ‘regenerated'. The proximal fragments that showed no change after 24 h were counted as ‘no regeneration'. A minimum of 50 individuals with 1–3 axotomized commissures were observed for most experiments. For PLM touch neurons, extended process lengths were measured from maximum transparency projections of a single z-stack, by using the Keyence BZ-X700 analysis software.

### Drug experiments

5-HT and forskolin were obtained from Wako and Focus Biomolecules, respectively. For 5-HT treatment, worms were grown from egg to adult, up until axotomy, on NGM plates containing 6.5 mM 5-HT and OP50 *Escherichia coli*. After axotomy, the worms were plated on NGM plates containing 5-HT and OP50 *E. coli* for 24 h. For forskolin treatment, the mixed stages of worms were incubated with M9 buffer containing 100 μm forskolin and heat-killed OP50 for 12 h at 20 °C, and the worms at young adult stage after the treatment were used.

### Immunofluorescence

5-HT immunofluorescence was essentially performed as described previously^5^. Briefly, the worms axotomized either D neurons or PLM neurons were fixed by 4% formaldehyde in PBS buffer at 4 °C for overnight, washed three times by PBS and then treated by PBS containing 5% β-mercaptoethanol and 1% Triton X-100 for 24 h. Then, the worms were washed by PBS and digested in 100 mM Tris-HCl (pH7.5), 1 mM CaCl_2_ and 5 mg ml^−1^ collagenase (Wako, 034–10533). After digestion, a rat anti-serotonin antibody (Chemicon, Cat. No. MAB352), 100X diluted with Can Get Signal Immunostain (TOYOBO), was used as the first antibody solution. A chicken anti-rat Alexa 594 (Molecular probes), 200X diluted with Can Get Signal Immunostain (TOYOBO), was used as the second antibody solution.

### Microscopy

Standard fluorescence images of transgenic worms were observed and photographed, using either a Zeiss Plan-APOCHROMAT × 100 objective of a Zeiss Axioplan II fluorescence microscope with a Hamamatsu 3CCD camera, or a × 100 objective of a Keyence BZ-X700 all-in-one fluorescence microscope. Confocal fluorescence images were taken on an Olympus FV500 and FV1000 confocal laser scanning microscopes with × 60 and × 100 objectives.

### Quantification of GFP expression

Expression of GFP fluorescence was quantified using the ImageJ program (NIH). The cell bodies of severed D or PLM neurons were outlined with closed polygons and the fluorescence intensities within these areas were determined (*I*_s_). Then *I*_s_ was divided by the area to derive intensity per area (*I*_sa_). To determine the background intensity of each cell, the same polygon was placed in an area neighbouring the cell body, fluorescence was measured (*I*_b_), and this was divided by area (*I*_ba_). The relative signal intensity per area (*I*_ra_) was calculated as *I*_sa_−*I*_ba_. Cells having *I*_ra_>7 (for D neuron) or *I*_ra_>4 (for PLM neuron) were categorized as ‘expressed', respectively.

### Statistical analysis

Statistical analyses were carried out as described previously^58^. Briefly, confidence intervals (95%) were calculated by the modified Wald method and two-tailed *P* values were calculated using Fisher's exact test (http://www.graphpad.com/quickcalcs/). The Welch's *t* -test was performed by using a *t*-test calculator (http://www.graphpad.com/quickcalcs/ttest1/).

## Additional information

**How to cite this article:** Alam, T. *et al.* Axotomy-induced HIF-serotonin signalling axis promotes axon regeneration in *C. elegans*. *Nat. Commun.* 7:10388 doi: 10.1038/ncomms10388 (2016).

## Supplementary Material

Supplementary Information Supplementary Figures 1-5 and Supplementary Tables 1-3.
