# Supplementary material for: Axotomy-induced HIF-serotonin signalling axis promotes axon regeneration in C. elegans
Source: Nat Commun. 2016 Jan 21;7:10388. doi: 10.1038/ncomms10388 (PMC4735912; doi:10.1038/ncomms10388)
Supplement: Supplementary Information — Supplementary Figures 1-5 and Supplementary Tables 1-3. [file ncomms10388-s1.pdf]

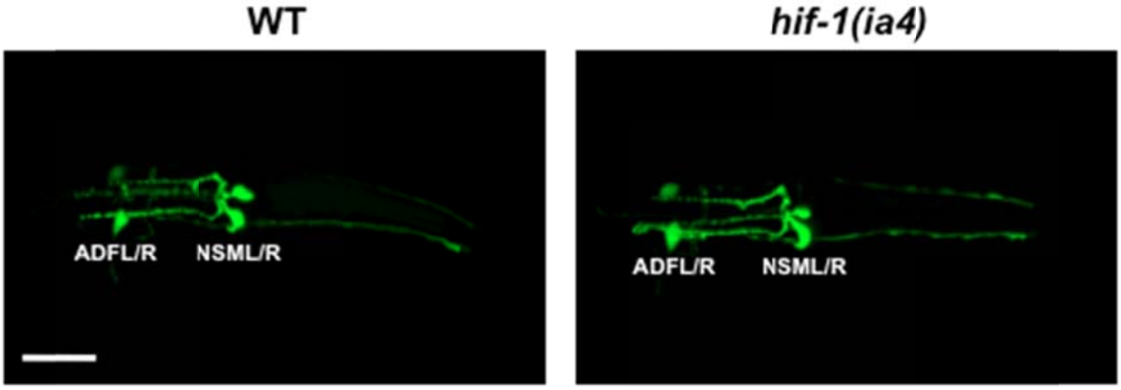

Supplementary Figure 1: *Ptph-1::gfp* expression in the head of animals
Ptph-1::gfp was expressed in NSM and ADF neurons in wild-type and *hif-1* mutant animals. Anterior is left. Scale bar = 20 μm.

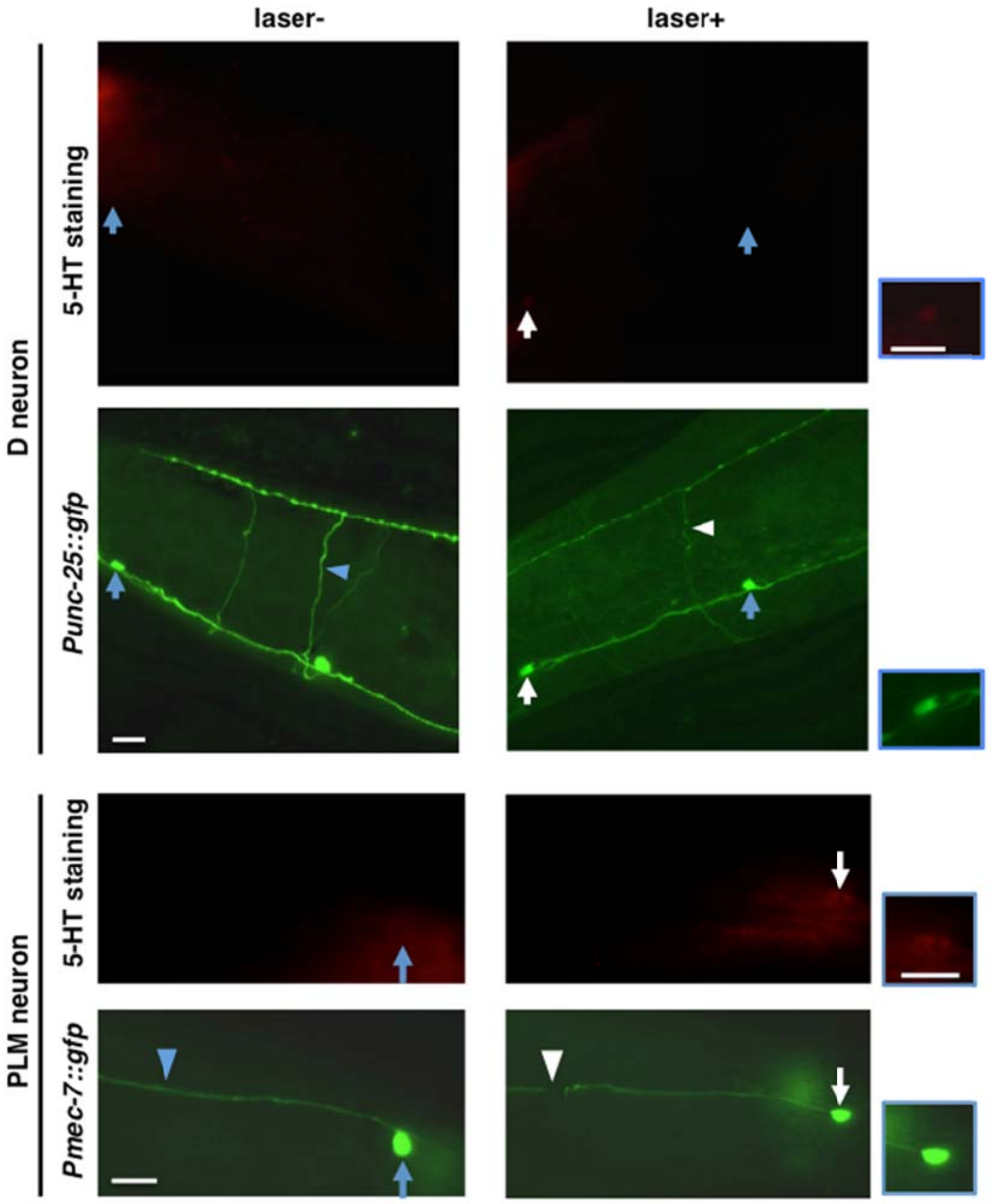

Supplementary Figure 2: 5-HT expression in D-type motor neurons and PLM sensory neurons following laser surgery
5-HT immunofluorescence in D-type motor neurons (row 1) and PLM neurons (row 3) 30 min after laser surgery is shown. Arrowheads and arrows indicate axons and cell bodies of D neurons and PLM neurons with (white) or without (blue) laser surgery, respectively. Both severed and unsevered PLM neurons within a same animal are shown. D neurons (row 2) and PLM neurons (row4) are visualized by GFP under control of the *unc-25* promoter and *mec-7* promoter, respectively. Cell bodies of severed neurons are magnified (blue boxes). Scale bars = 20 μm.

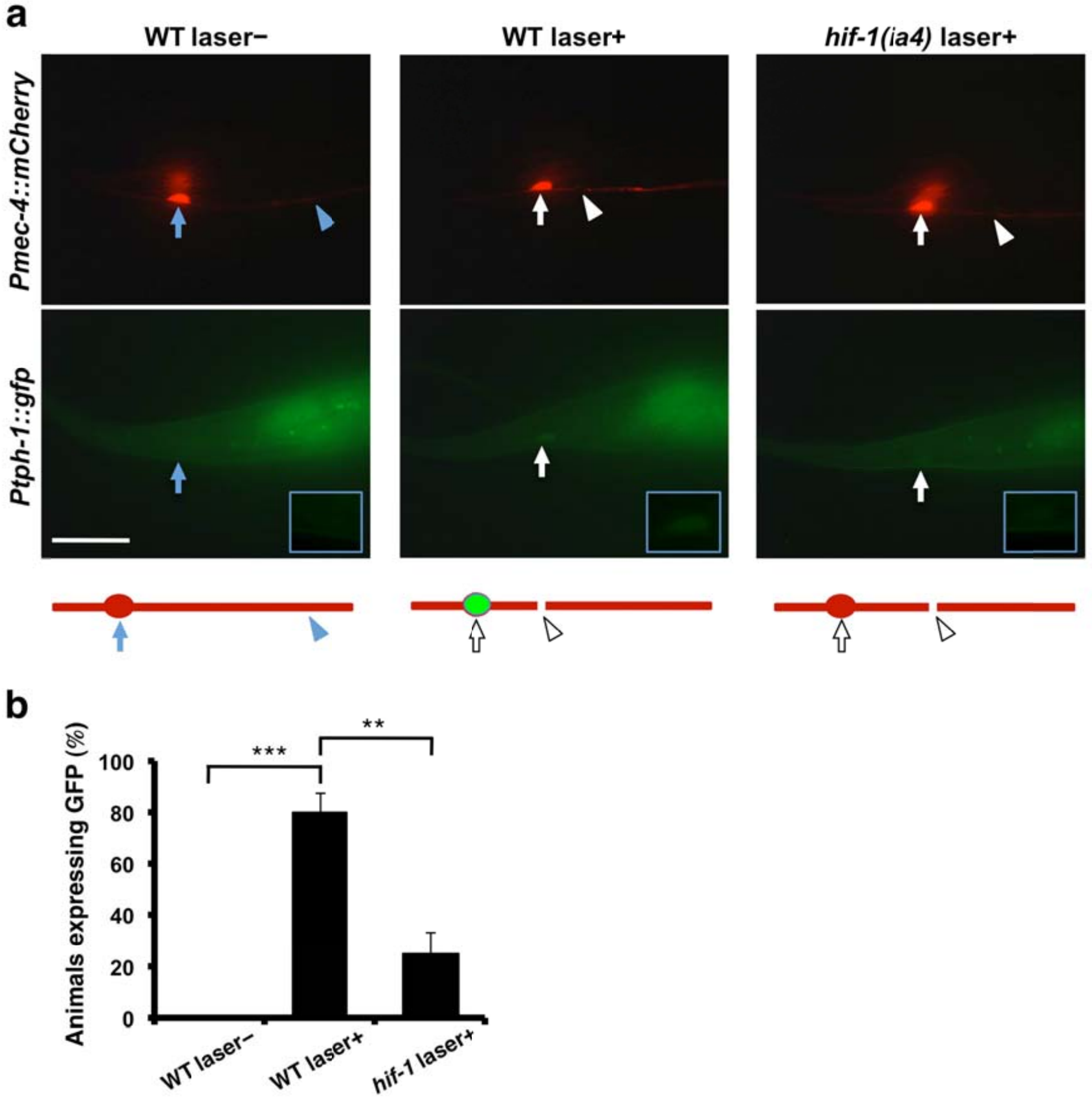

Supplementary Figure 3: Induction of *Ptph-1::gfp* expression in PLM sensory neurons by laser surgery

(**a**) Induction of *Ptph-1::gfp* expression in PLM sensory neurons by laser surgery. Expression of fluorescent proteins in PLM sensory neurons 30 min after laser surgery is shown. A schematic representation of PLM sensory neurons is shown on the bottom. Arrowheads and arrows indicate axons and cell bodies of PLM neurons with (white) or without (blue) laser surgery, respectively. PLM neurons are visualized by mCherry under control of the *mec-4* promoter. Cell bodies of PLM neurons are magnified (blue boxes). Scale bar = 20 μm.

(**b**) Percentages of animals expressing *tph-1*. Induction of *Ptph-1::gfp* expression in PLM sensory neurons with (+) or without (-) laser surgery was assayed as described in Methods. Twenty neurons were examined for each condition. Error bars indicate 95% CI. ***$P<0.001$; **$P<0.01$ (Fisher's exact test, two-tailed, n≥50).

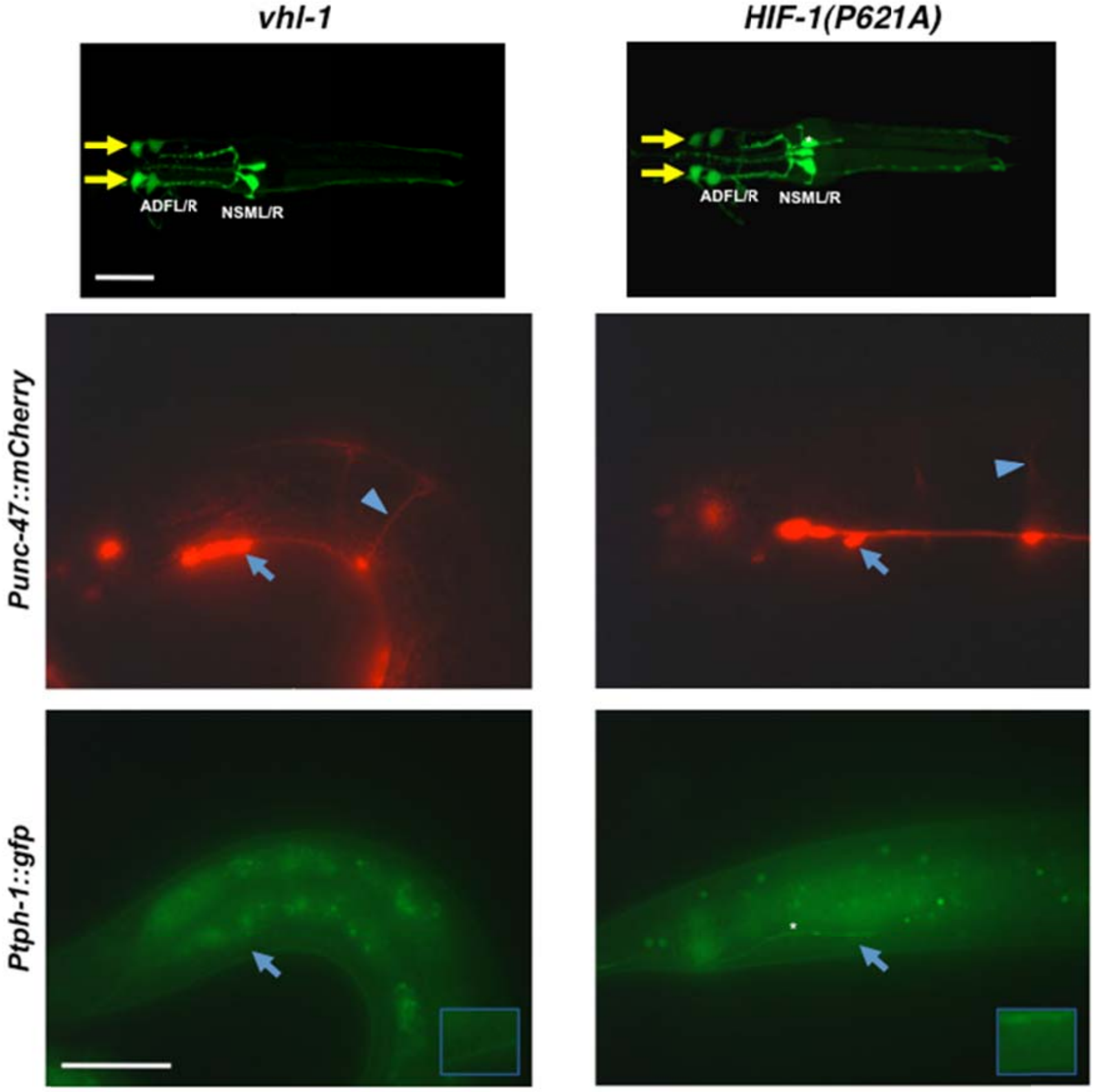

Supplementary Figure 4: Effect of HIF-1 stabilization on *Ptph-1::gfp* expression

Expression of fluorescent proteins in ASG neurons and D-type motor neurons is shown. ASG neurons are indicated by yellow arrows. D neurons are visualized by mCherry under control of the *unc-47* promoter. Blue arrowheads and arrows indicate axons and cell bodies of D-type neurons, respectively. Cell bodies of D-type neurons are magnified (blue boxes). Asterisks indicate ectopic expression in MC and PHA neurons observed in HIF-1(P621A)-expressing animals. Scale bars = 20 μm.

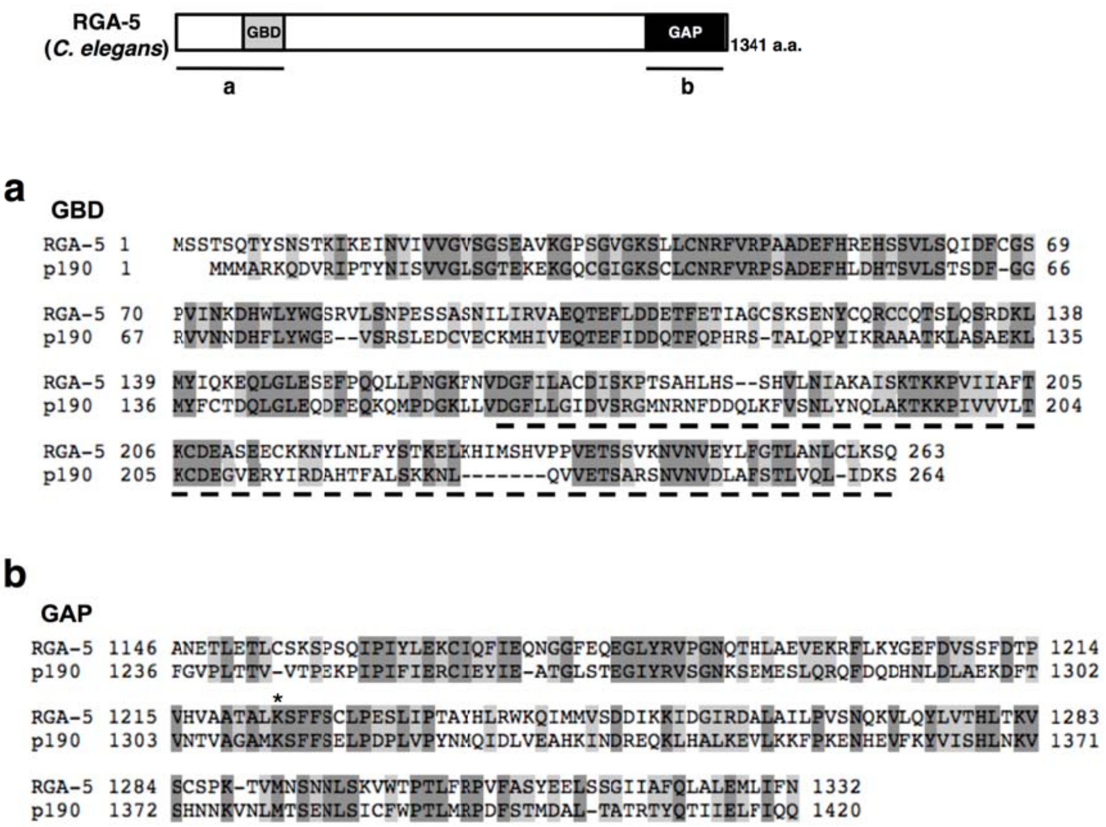

Supplementary Figure 5: Comparisons of domains between mammalian p190 RhoGAP and *C. elegans* RGA-5

(**a** , **b**) The GTP binding (**a**) and GAP (**b**) domains are shown. Identical and similar residues are highlighted with black and gray shading, respectively. GBD domains are indicated by a dashed line. The conserved Lys residue essential for GAP activity is indicated by an asterisk.

Strain	Genotype	No. of animals	No. of axons	Regrowth (μm ± SEM)	P vs. control*
KU1343	*muIs32*	32	32	79.2 ± 4.6	-
KU1344	*tph-1(mg280) muIs32*	30	30	51.8 ± 4.1	<0.001
KU1347	*hif-1(ia4); muIs32*	30	30	56.2 ± 4.7	<0.001

* unpaired *t* -test, two-tailed, unequal variances.

Supplementary Table 1: Raw data for genotypes tested by axotomy

Strain	Genotype	No. of animals	No. of axons	No. of regeneration	*P* vs. control*
KU501	*juIs76*	58	78	55 (71%)	-
KU1317	*tph-1(mg280) juIs76*	25	53	18 (34%)	<0.001
KU1329	*tph-1(n4622) juIs76*	26	52	18 (35%)	<0.001
KU1317	*tph-1(mg280) juIs76* + serotonin	29	60	36 (60%)	0.0081 [e]
KU1319	*tph-1(mg280) juIs76; Punc-25::tph-1*	23	52	30 (58%)	0.019 [e]
KU1330	*tph-1(mg280) juIs76; Pmec-7::tph-1*	27	52	25 (48%)	0.17 [e]
KU1331	*tph-1(mg280) juIs76; Psrh-142::tph-1*	24	51	18 (35%)	1.0 [e]
KU1320	*tph-1(mg280) juIs76; floxed tph-1*	28	59	37 (63%)	0.0027 [e]
KU1321	*tph-1(mg280) juIs76; floxed tph-1; Psrh-142::nls-cre*	23	50	28 (56%)	0.56 [f]
KU1322	*tph-1(mg280) juIs76; floxed tph-1; Pceh-2::nls-cre*	24	52	35 (67%)	0.69 [f]
KU1323	*tph-1(mg280) juIs76; floxed tph-1; Pegl-6::nls-cre*	24	50	32 (64%)	1.0 [f]
KU1324	*tph-1(mg280) juIs76; floxed tph-1; Punc-25::nls-cre*	24	50	19 (38%)	0.013 [f]
KU1327	*hif-1(ia4); juIs76*	24	51	19 (37%)	<0.001
KU1332	*hif-1(ok2564); juIs76*	28	50	13 (26%)	<0.001
KU1327	*hif-1(ia4); juIs76* + serotonin	31	64	38 (59%)	0.024 [g]
KU1313	*ser-1; juIs76*	27	50	31 (62%)	0.34
KU1314	*ser-7(tm1325); juIs76*	34	52	16 (31%)	<0.001
KU1333	*ser-7(tm1548); juIs76*	30	53	16 (30%)	<0.001
KU1314	*ser-7(tm1325); juIs76* +serotonin	26	55	26 (47%)	0.11 [d]
KU1315	*ser-7(tm1325); juIs76; Punc-25::ser-7*	21	52	27 (52%)	0.046 [d]
KU1316	*ser-7(tm1325); juIs76; Pmec-7::ser-7*	25	52	18 (35%)	0.84 [d]
KU1310	*gpa-12(pk322); juIs76*	33	51	21 (41%)	0.0017
KU1334	*gpa-12(gk766855); juIs76*	30	52	16 (31%)	<0.001
KU1335	*ser-7(tm1325) gpa-12(pk322); juIs76*	22	51	19 (37%)	0.84 [c]
KU1303	*rhgf-1(ok880); juIs76*	30	55	20 (36%)	<0.001
KU1336	*rhgf-1(gk217); juIs76*	29	52	18 (35%)	<0.001
KU1304	*rhgf-1(ok880); juIs76; Punc-25::rho-1(G14V)*	32	50	33 (66%)	0.0033 [a]
KU1305	*rhgf-1(ok880); juIs76; Punc-25::rho-1(T19N)*	29	63	20 (32%)	0.70 [a]
KU1301	*rga-5; juIs76*	25	50	39 (78%)	0.41
KU1302	*juIs76; Punc-25::rga-5gap*	33	59	18 (31%)	<0.001
KU1337	*juIs76; Punc-25::rga-5gap(K1223A)*	24	52	34 (65%)	0.57
KU1306	*rga-5; rhgf-1(ok880); juIs76*	35	50	29 (58%)	0.032 [a]
KU1307	*dgk-1; juIs76*	28	50	31 (62%)	0.34
KU1308	*dgk-1 rhgf-1(ok880); juIs76*	36	61	36 (59%)	0.017 [a]
KU1309	*dgk-1; juIs76; Punc-25::rga-5gap*	29	59	34 (58%)	0.0052 [b]
KU1311	*rga-5; gpa-12(pk322); juIs76*	29	60	37 (62%)	0.037 [c]
KU1312	*gpa-12(pk322) dgk-1; juIs76*	33	60	37 (62%)	0.037 [c]
KU1338	*rga-5; ser-7(tm1325); juIs76*	32	59	22 (37%)	0.55 [d]
KU1339	*ser-7(tm1325) dgk-1; juIs76*	27	52	20 (38%)	0.54 [d]
KU1314	*ser-7(tm1325); juIs76* + forskolin	30	62	22 (35%)	0.69 [d]
KU1338	*rga-5; ser-7(tm1325); juIs76* + forskolin	26	62	32 (52%)	0.036 [d]
KU1340	*juIs76; Punc-25::pde-4*	31	61	28 (46%)	0.0051
KU1341	*acy-1; juIs76*	32	64	21 (33%)	<0.001
KU1342	*acy-1; ser-7(tm1325); juIs76*	22	50	17 (34%)	1.0 [h]

*Fisher's exact test, two-tailed.
a: KU1303 as a control, b: KU1302 as a control, c: KU1310 as a control, d: KU1314 as a control,
e: KU1317 as a control, f: KU1320 as a control, g: KU1327 as a control, h: KU1341 as a control.

Supplementary Table 2: Raw data for genotypes tested by axotomy

**Strain**	**Genotype**
KU1343	*muIs32 Ⅱ.*
KU1344	*tph-1(mg280) muIs32 Ⅱ.*
KU501	*juIs76 Ⅱ.*
KU1317	*tph-1(mg280) juIs76 Ⅱ.*
KU1329	*tph-1(n4622) juIs76 Ⅱ.*
KU1319	*tph-1(mg280) juIs76 Ⅱ; kmEx1307.*
KU1330	*tph-1(mg280) juIs76 Ⅱ; kmEx1310.*
KU1331	*tph-1(mg280) juIs76 Ⅱ; kmEx1311.*
KU1320	*tph-1(mg280) juIs76 Ⅱ; kySi56.*
KU1321	*tph-1(mg280) juIs76 Ⅱ; kySi56; kyEx4077.*
KU1322	*tph-1(mg280) juIs76 Ⅱ; kySi56; kyEx4057.*
KU1323	*tph-1(mg280) juIs76 Ⅱ; kySi56; kyEx4107.*
KU1324	*tph-1(mg280) juIs76 Ⅱ; kySi56; kmEx1308.*
KU1325	*zdIs13 Ⅳ; kmEx1309*
KU1326	*zdIs13 Ⅳ; hif-1(ia4) Ⅴ; kmEx1309*
KU1345	*wpIs36 Ⅰ; zdIs13 Ⅳ.*
KU1346	*wpIs36 Ⅰ; zdIs13 Ⅳ; hif-1(ia4) Ⅴ.*
KU1327	*juIs76 Ⅱ; hif-1(ia4) Ⅴ.*
KU1332	*juIs76 Ⅱ; hif-1(ok2564) Ⅴ.*
KU1347	*muIs32 Ⅱ ; hif-1(ia4) Ⅴ.*
KU1313	*juIs76 Ⅱ; ser-1(ok345)X.*
KU1314	*juIs76 Ⅱ; ser-7(tm1325)X.*
KU1333	*juIs76 Ⅱ; ser-7(tm1548)X.*
KU1315	*juIs76 Ⅱ; ser-7(tm1325)X; kmEx1304.*
KU1316	*juIs76 Ⅱ; ser-7(tm1325)X; kmEx1305.*
KU1310	*juIs76 Ⅱ; gpa-12(pk322)X.*
KU1334	*juIs76 Ⅱ; gpa-12(gk766855)X.*
KU1335	*juIs76 Ⅱ; ser-7(tm1325) gpa-12(pk322)X.*
KU1303	*juIs76 Ⅱ; rhgf-1(ok880)X.*
KU1336	*juIs76 Ⅱ; rhgf-1(gk217)X.*
KU1304	*juIs76 Ⅱ; rhgf-1(ok880)X; kmEx1302.*
KU1305	*juIs76 Ⅱ; rhgf-1(ok880)X; kmEx1303.*
KU1301	*juIs76 Ⅱ; rga-5(ok2241)Ⅳ.*
KU1302	*juIs76 Ⅱ; kmEx1301.*
KU1337	*juIs76 Ⅱ; kmEx1312.*
KU1306	*juIs76 Ⅱ; rga-5(ok2241)Ⅳ; rhgf-1(ok880)X.*
KU1307	*juIs76 Ⅱ; dgk-1(ok1462)X.*
KU1308	*juIs76 Ⅱ; dgk-1(ok1462) rhgf-1(ok880)X.*
KU1309	*juIs76 Ⅱ; dgk-1(ok1462)X; kmEx1301.*
KU1311	*juIs76 Ⅱ; rga-5(ok2241)Ⅳ; gpa-12(pk322)X.*
KU1312	*juIs76 Ⅱ; gpa-12(pk322) dgk-1(ok1462)X.*
KU1338	*juIs76 Ⅱ; rga-5(ok2241)Ⅳ; ser-7(tm1325)X.*
KU1339	*juIs76 Ⅱ; ser-7(tm1325) dgk-1(ok1462)X.*
KU1340	*juIs76 Ⅱ; kmEx1306.*
KU1341	*juIs76 Ⅱ; acy-1(nu329)Ⅲ.*
KU1342	*juIs76 Ⅱ; acy-1(nu329)Ⅲ; ser-7(tm1325)X.*
KU1328	*zdIs13 Ⅳ; wdEx848.*
KU1348	*zdIs13 Ⅳ; hif-1(ia4) Ⅴ; wdEx848.*
KU1349	*wpIs36 Ⅰ; zdIs13 Ⅳ; vhl-1(ok161) X.*
KU1350	*wpIs36 Ⅰ; zdIs13 otIs197 Ⅳ.*

Supplementary Table 3: Strains used in this study
